# Analyses on safety and efficacy of non-standard dose of r-tPA in intravenous thrombolysis-treated AIS patients

**DOI:** 10.3389/fneur.2022.1007167

**Published:** 2022-11-14

**Authors:** Jiawen Yuan, Ruxing Wu, Jingyan Xiang, Jiangshan Deng, Xiaojie Zhang, Kaili Lu, Fengya Cao, Fei Zhao, Yuwu Zhao, Feng Wang

**Affiliations:** ^1^Department of Neurology, Shanghai Sixth People's Hospital Affiliated to Shanghai Jiao Tong University School of Medicine, Shanghai, China; ^2^School of Nursing, Shanghai Jiao Tong University, Shanghai, China

**Keywords:** acute ischemic stroke, intravenous thrombolysis, r-tPA, dose, sub-group analyses

## Abstract

**Background:**

Intravenous 0.9 mg/kg recombinant tissue plasminogen activator (r-tPA) is one of the most effective treatments in acute ischemic stroke patients. Practically, the dose of r-tPA is still a topic that is constantly being discussed.

**Methods:**

For this observational study, data were obtained from 537 patients who received r-tPA thrombolysis at Shanghai Sixth People's Hospital stroke center over 5 years (2014–2019). Patients were divided into two groups: a non-standard dose group (0.6 mg/kg ≤ dose < 0.9 mg/kg) and a standard dose group (0.9 mg/kg). Different outcomes were observed: efficacy: 3 months mRS 0-1 (3m-mRS0-1); safety: symptomatic intracranial hemorrhage within 24 h (24h-sICH) and 3 months mortality (3m-death). We also observed the effect of r-tPA dose coefficient on outcomes in different age groups and baseline National Institute of Health stroke scale (NIHSS) score subgroups.

**Results:**

There were 265 patients who gave the standard dose treatment and 272 gave the nonstandard dose. There was no significant difference between the non-standard dose group and the standard dose group in 3m-mRS0-1, 3m-death, and 24h-sICH (*p* = 0.567, 0.327, and 0.415, respectively). The dose coefficient presents a significant negative correlation (*p* = 0.034, B = −4.290) with 3m-death in NIHSS < 16 sub-group. Door-to-needle time (DNT) is the most important independent outcome-influential factor (MIOIF) in the NIHSS ≥16 sub-group. The diabetes history and baseline NIHSS score were the MIOIF in the age ≥80-year sub-group.

**Conclusions:**

The non-standard dose group (0.6 mg/kg ≤ dose < 0.9 mg/kg) shows no difference in safety and effectiveness than the standard dose group (0.9 mg/kg) in our study. The standard dose should be considered first according to current evidence and Guidelines, but the non-standard dose (0.6 mg/kg ≤ dose < 0.9 mg/kg) might be an option in the actual diagnosis and treatment process considering the patient's clinical profile and financial condition.

## Introduction

Intravenous thrombolysis using recombinant tissue plasminogen activator (r-tPA) is widely used in acute ischemic stroke (AIS) patients (1, 2). In recent years in China, with the popularization of stroke knowledge, improvement of out-hospital emergency system, and coverage of r-tPA by medical insurance, the ratio of thrombolysis was raised to 24.2% in the year 2018 (3). The main constraints are still largely the patient's own cost and the fear of symptomatic intracranial hemorrhage (sICH).

In the real world, physicians, especially in many developing countries in Asia such as China, may choose r-tPA in a way that does not fully comply with the standard dose for different reasons, such as a patient's financial condition, advanced age, or the onset of a serious illness. Meanwhile, the dose of r-tPA is still a topic that is constantly being discussed. The Japan Alteplase Clinical Trial first demonstrated that AIS patients receiving r-tPA at a dose of 0.6 mg/kg could obtain comparable efficacy and safety to historical controls given 0.9 mg/kg r-tPA (4–8). While the ENCHANTED study (9) showed that compared with the standard dose, the lower dose group was not inferior in the ordinal analysis of modified Rankin scale scores (mRS), and with no significantly higher mortality at 90 days, while the sICH and fatal events occurred within 7 days are reduced. Beside 0.6 mg/kg, other low doses of IV r-tPA have been studied in specific values. In 2017, Cheung-Ter Ong et Al. further compared the efficacy and safety of different doses (0.6, 0.7, 0.8, and 0.9 mg/kg) of r-tPA (10). The early neurological improvement, early neurological deterioration, and the sICH were not significantly different among the four dosage groups. But the clinical functional outcome at 6 months after stroke onset was poorer than in the standard-dose group (*P* = 0.02). All these trials pre-assigned the dose (mg/kg) in a fixed value, but what is the relationship between the doses (as a continuous variable) and the outcome of AIS patients treated with r-tPA? In fact, in clinical practice, many factors affect the prognosis of AIS patients in the guidelines published by AHA/ASA for the Early Management of Acute Ischemic Stroke (1) and the Chinese guidelines for clinical management of ischemic cerebrovascular disease (11). Besides, the idea that thrombolysis should be initiated as quickly as possible because timely treatment is strongly associated with better outcomes, other factors like age and NIHSS score before thrombolysis are also vital factors that affect the outcome. In detail, when the onset to thrombolysis time (OTT) is < 3 h, IV alteplase administration is equally recommended for patients ≤ 80 and >80 years of age, and also it is recommended for patients with severe stroke and with mild but disabling stroke symptoms (1, 11). When OTT is within 3 and 4.5 h, those patients >80 years of age or with severe stroke symptoms (NIHSS > 25), the benefit of IV alteplase is uncertain or lower dose alteplase (0.6 mg/kg) can be given as an alternative (11). Therefore, there are still uncertain opinions on the dose of r-tPA in patients with different major characteristics (like age and NIHSS onset).

In this study, we observed 537 patients with r-tPA treatment in the year from 2014 to 2019 (data from the real world). There are patients given nonstandard dose r-tPA treatment and standard dose r-tPA treatment. We compared the effect of grouping on the outcome. Age subgroups and NIHSS subgroups were also analyzed to find the relationship between the drug coefficient and the outcome of r-tPA-treated AIS patients.

## Materials and methods

This retrospective observational study protocol was carried out according to the recommendations of the Ethics Committee of Shanghai Jiao Tong University Affiliated Sixth People's Hospital and was registered in the Chinese Clinical Trial Registry (accession number: ChiCTR1900024521). All the subjects gave written informed consent according to the Declaration of Helsinki.

### Participants and procedures

We have a database for patients who received r-tPA intravenous thrombolysis in our hospital since 2010, including the general situation of the patient, neurological function scores at various stages, laboratory examination results, imaging results, follow-up results, and so on. Our center is one of the 12 municipal stroke treatment centers, which participated in the quality-improvement project for stroke care throughout Shanghai (population of more than 20 million). There are about 100 cases of intravenous thrombolysis every year, about 30 cases can enter the green channel of stroke intervention every month, and about 33% of patients can receive intravenous thrombolytic therapy. About 50% of patients have a DNT time of < 60 min, and 25% have a DNT time of < 45 min. The incidence of sICH is about 4%. The proportion of patients with good prognosis (mRS score 0–1) is about 57%.

All patients treated with r-tPA met the standard of the scientific statement of the Chinese Stroke Society on intravenous thrombolysis in acute ischemic stroke (12). In our study, we enrolled 537 patients with complete baseline information and follow-up data (21 patients lost to follow-up within 3 months and 14 patients with incomplete key data) from the year 2014 to 2019. Two hundred and sixty-five patients accepted r-tPA with a dose of 0.9 mg/kg, while 272 patients received a lower dose (≥0.6 mg/kg and < 0.9 mg/kg) for kinds of reasons (mentioned above). Our team recorded the features of patients, including gender, age, OTT, weightdose of drug, systolic blood pressure (SBP), diastolic blood pressure (DBP), admission glucose, baseline National Institute of Health stroke scale (NIHSS) score, history of hypertension (HTN), history of diabetes mellitus (DM), history of coronary atherosclerotic heart disease (CHD), history of atrial fibrillation (Af), previous stroke or transient ischemic attack (TIA), and followed up 24h sICH and 3-month mRS. All the recorded variables between the two groups were compared. The correlation between the outcomes and these variables (including grouping) was analyzed. Furthermore, patients were divided into different subgroups according to age (≥80 years or < 80 years) and NIHSS score before thrombolysis (≥16 or < 16). In each subgroup, we studied the correlations between the outcomes and variables.

### Statistical analyses

The chi-square test and independent-sample t-test were used for the comparison of baseline data between the standard dose group and the non-standard dose group. In the dose grouping analyses, we also used the chi-square test for categorical variables and the independent-sample t-test for continuous variables, as single factor analysis. Multivariate logistic regression analysis was sequentially performed for further ascertaining the outcome-influential factors, after adjusting for potential confounders (group, age, history of atrial fibrillation to 3m-mRS0-1; group, age, DBP to 24h-sICH; group, history of coronary heart disease to 3m-death). In the age sub-groups and NIHSS sub-groups analyses, chi-square test was also used for studying the relationship between categorical variables to the outcomes, while Mann–Whitney Rank Sum Test was used for testing the continuous variables (not conformed to normal distribution), respectively. Multivariate logistic regression analysis was sequentially used for further ascertaining the outcome-influential factors, after adjusting for potential confounders. The dose coefficient was ascertained through multiple regression analysis, whether it has a significant effect on the outcome after univariate analysis or not.

## Results

### Comparison of baseline data between standard and non-standard dose groups

From 2014 to 2019, a total of 537 AIS patients received r-tPA. A total of 265 patients accepted the standard r-tPA dose of 0.9 mg/kg, while 272 patients received the non-standard dose (≥0.6 mg/kg and < 0.9 mg/kg). The comparison of baseline data between the two groups is shown in Table 1. Gender, OTT, and body weight are significantly different between the two groups (*p* = 0.002, 0.016 and 0.001, respectively).

**Table 1:** General information on non-standard group and standard group patients.

	**All samples**	**Non-standard group**	**Standard group**	***χ^2^*/*t* value**	***P***
	**(*n* = 537)**	**(*n* = 272)**	**(*n* = 265)**		
**Gender**					
Male	366 (68.16)	202 (74.26)	164 (61.89)	9.48	0.002
Female	171 (31.84)	70 (25.74)	101 (38.11)		
**Age (year)**	65.39 ± 11.62	65.44 ± 11.93	65.33 ± 11.33	0.11	0.916
**OTT (min)**	176.33 ± 55.78	182.02 ± 56.85	170.48 ± 54.14	2.41	0.016
**DNT (min)**	74.94 ± 32.76	76.60 ± 34.12	73.24 ± 31.28	1.19	0.235
**Weight (kg)**	67.93 ± 11.08	69.53 ± 9.77	66.30 ± 12.09	3.40	0.001
**SBP (mmHg)**	149.35 ± 20.50	149.53 ± 20.70	149.18 ± 20.32	0.20	0.844
**DBP (mmHg)**	82.74 ± 12.79	83.17 ± 12.37	82.31 ± 13.21	0.77	0.439
**Admission glucose (mmol/L)**	7.66 ± 3.31	7.46 ± 3.14	7.87 ± 3.47	−1.44	0.150
**History of hypertension (Yes)**	341 (63.50)	182 (66.91)	159 (60.00)	2.77	0.096
**Diabetes history (**Yes)	111 (20.67)	54 (19.85)	57 (21.51)	0.23	0.636
**History of coronary heart disease (Yes)**	29 (5.40)	17 (6.25)	12 (4.53)	0.78	0.377
**History of atrial fibrillation (Yes)**	120 (22.35)	60 (22.06)	60 (22.64)	0.03	0.871
**History of Stroke/TIA (Yes)**	68 (12.66)	37 (13.60)	31 (11.70)	0.44	0.507
**NIHSS-before thrombolysis**	9.10 ± 6.57	9.03 ± 6.69	9.18 ± 6.47	−0.26	0.795
**r-tPA Dose/kg**	0.84 ± 0.08	0.79 ± 0.08	0.90 ± 0.01	−24.53	< 0.001

OTT, onset to thrombolysis time; DNT, door to needle time; SBP, systolic blood pressure; DBP, diastolic blood pressure; NIHSS, National Institute of Health stroke scale.

After equation stepping, the independent influencing factors of each outcome (3m-mRS0-1, 3m-death, and 24h-sICH) were further confirmed in Table 2. We found that OTT, DNT, SBP, baseline Glucose, and NIHSS score had an independent significant effect on 3m-mRS0-1 (*p* = 0.007, 0.016, 0.004, 0.018, and < 0.001, respectively). Baseline Glucose, history of fibrillation, SBP, and NIHSS score were significantly related to 24h-sICH (*p* = 0.01, < 0.001, 0.003, and < 0.001, respectively). Age, DNT, SBP, history of fibrillation, and NIHSS score have significant effect on 3m-death (*p* = 0.023, 0.003, 0.013, 0.022, and < 0.001, respectively). Besides these factors, group discrimination was not found to be an independent influencing factor of any outcome index (3m-mRS0-1, *p* =0.314; 24h-sICH, *p* =0.109; and 3m-death, *p* =0.196, respectively).

**Table 2:** Independent influencing factors of each outcome in total patients.

**Independent variables**	**β**	**S.E**.	**Wald χ^2^**	***p*-value**	**OR (95%CI)**
**3m-mRS0-1**					
OTT (min)	−0.005	0.002	7.181	0.007	0.995 (0.991, 0.999)
DNT (min)	−0.008	0.003	5.814	0.016	0.992 (0.985, 0.998)
SBP (mmHg)	−0.015	0.005	8.278	0.004	0.985 (0.975, 0.995)
Admission glucose (mmol/L)	−0.074	0.031	5.580	0.018	0.928 (0.873, 0.987)
NIHSS-before thrombolysis	−0.198	0.020	100.174	< 0.001	0.820 (0.789, 0.852)
**24h-sICH**					
History of atrial fibrillation (Yes)	1.692	0.449	14.225	< 0.001	5.429 (2.254, 13.077)
SBP (mmHg)	0.038	0.013	8.771	0.003	1.038 (1.013, 1.064)
Admission glucose (mmol/L)	0.124	0.048	6.550	0.010	1.132 (1.029, 1.244)
NIHSS-before thrombolysis	0.131	0.032	16.431	< 0.001	1.140 (1.070, 1.215)
**3m-death**					
History of atrial fibrillation (yes)	0.759	0.330	5.273	0.022	2.136 (1.118, 4.081)
Age (year)	0.036	0.016	5.160	0.023	1.036 (1.005, 1.069)
DNT (min)	0.013	0.004	8.615	0.003	1.013 (1.004, 1.022)
SBP (mmHg)	0.020	0.008	6.110	0.013	1.020 (1.004, 1.037)
Admission glucose (mmol/L)	0.078	0.040	3.752	0.053	1.081 (0.999, 1.169)
NIHSS-before thrombolysis	0.148	0.023	41.934	< 0.001	1.160 (1.109, 1.213)

OTT, onset to thrombolysis time; DNT, door to needle time; SBP, systolic blood pressure; DBP, diastolic blood pressure; mRS, modified Rankin scale; sICH, symptomatic intracranial hemorrhage; NIHSS, National Institute of Health stroke scale.

### Sub-group analyses according to baseline NIHSS score

To study the prognosis of acute ischemic stroke patients with different clinical characteristics using thrombolytic therapy more accurately, and to confirm the correlation between drug dose coefficient and prognosis more directly, we conducted sub-group analyses.

**Figure 1:**
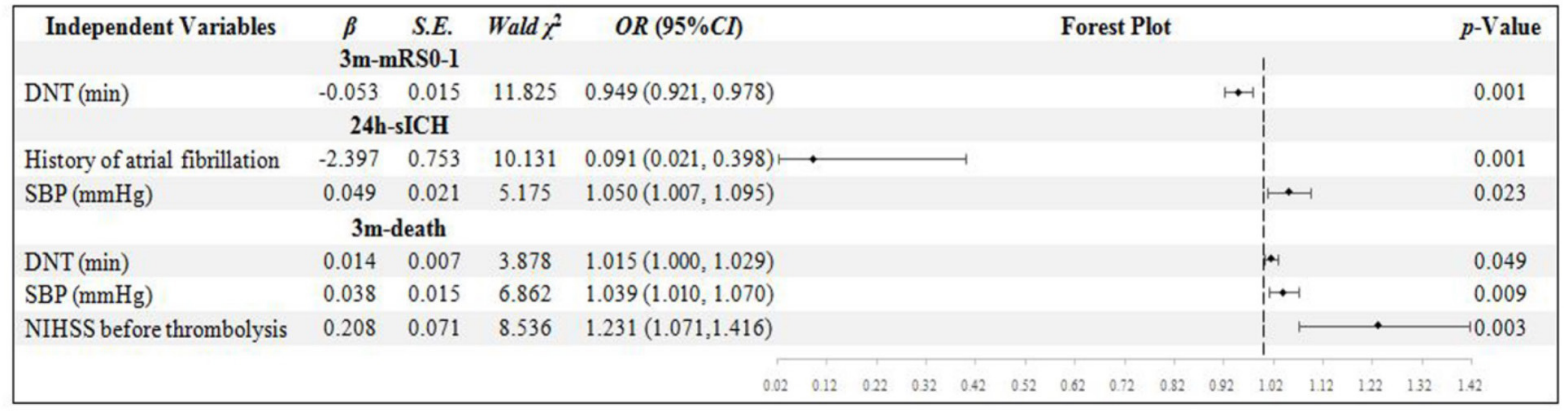
Binary logistic regression analysis in NIHSS score ≥ 16 group. DNT, door to needle time; SBP, systolic blood pressure; NIHSS, National Institute of Health Stroke Scale; mRS, modified Rankin scale; sICH, symptomatic intracranial hemorrhage.

In the NIHSS score ≥16 sub-group, 92 patients' records were analyzed. Only DNT was confirmed to be the independent influential factor to 3m-mRs0-1 (*p* = 0.001). History of atrial fibrillation and SBP were confirmed to be the independent influential factor to 24h-sICH (*p* = 0.001 and 0.023). The DNT, SBP, and NIHSS scores were confirmed to be the independent influential factor to 3m-death (*p* = 0.049, 0.009, and 0.003, respectively) (Figure 1).

**Figure 2:**
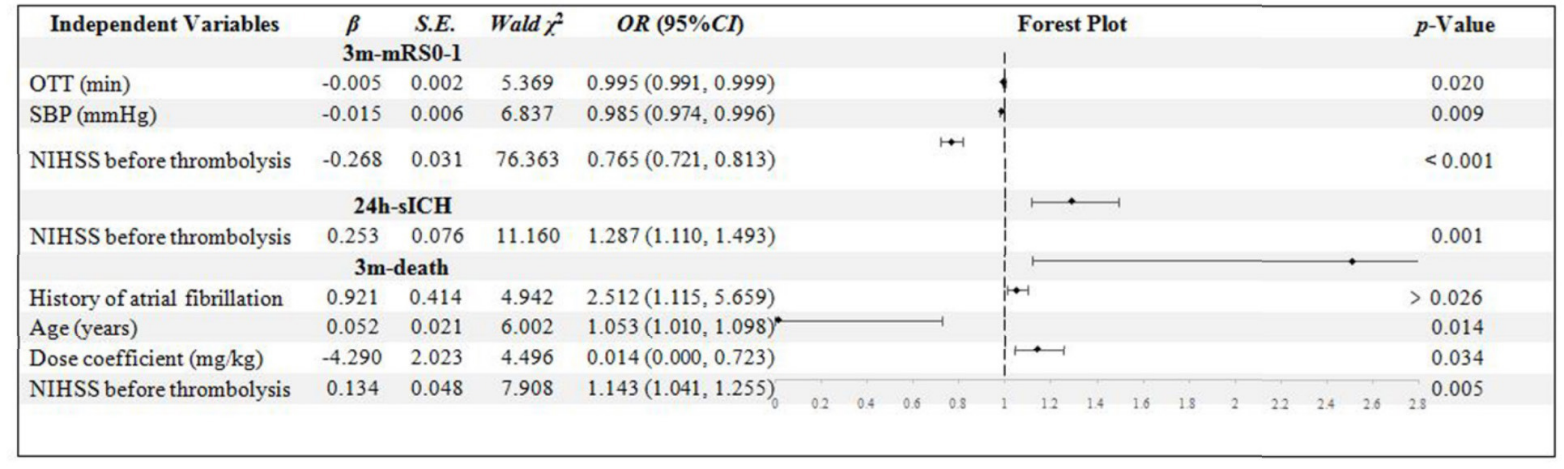
Binary logistic regression analysis in NIHSS score < 16 group. OTT, onset to thrombolysis time; SBP, systolic blood pressure; NIHSS, National Institute of Health stroke scale; mRS, modified Rankin scale; sICH, symptomatic intracranial hemorrhage.

In the NIHSS score < 16 group, 445 patients were included. The OTT, SBP, and NIHSS score were confirmed to be the independent influential factors to 3m-mRs0-1 (*p* = 0.020, 0.009, and < 0.001, respectively). Only the NIHSS score was confirmed to be the independent influential factor to 24h-sICH (*p* = 0.001). History of Af, age, NIHSS score, and dose coefficient was confirmed to be the independent influential factors to 3m-death (*p* = 0.026, 0.014, 0.034, and 0.005, respectively). The higher dose coefficient was related to the less possibility of 3m-death (Figure 2).

### Sub-group analyses according to age

**Figure 3:**
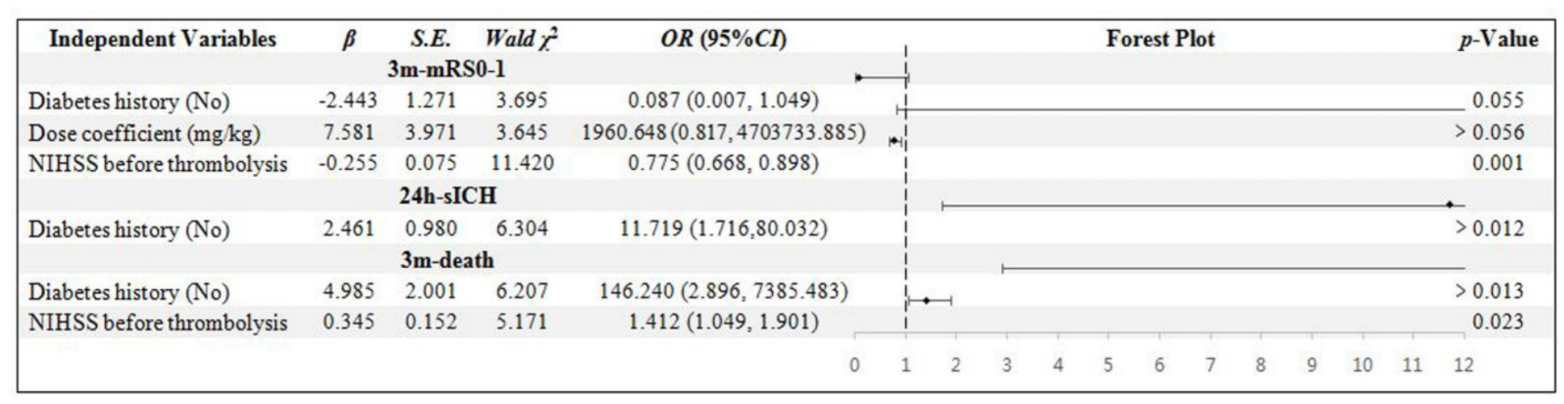
Binary logistic regression analysis in age ≥ 80 years group. NIHSS, National Institute of Health stroke scale; mRS, modified Rankin scale; sICH, symptomatic intracranial hemorrhage.

There are 51 patients in the age ≥80 years group. The NIHSS score was confirmed to be the independent influential factor to 3m-mRs0-1 and 3m-death (*p* = 0.001 and 0.023). The diabetes history was confirmed to be related to the 24h-sICH and 3m-death independently (*p* = 0.012 and 0.013) (Figure 3).

**Figure 4:**
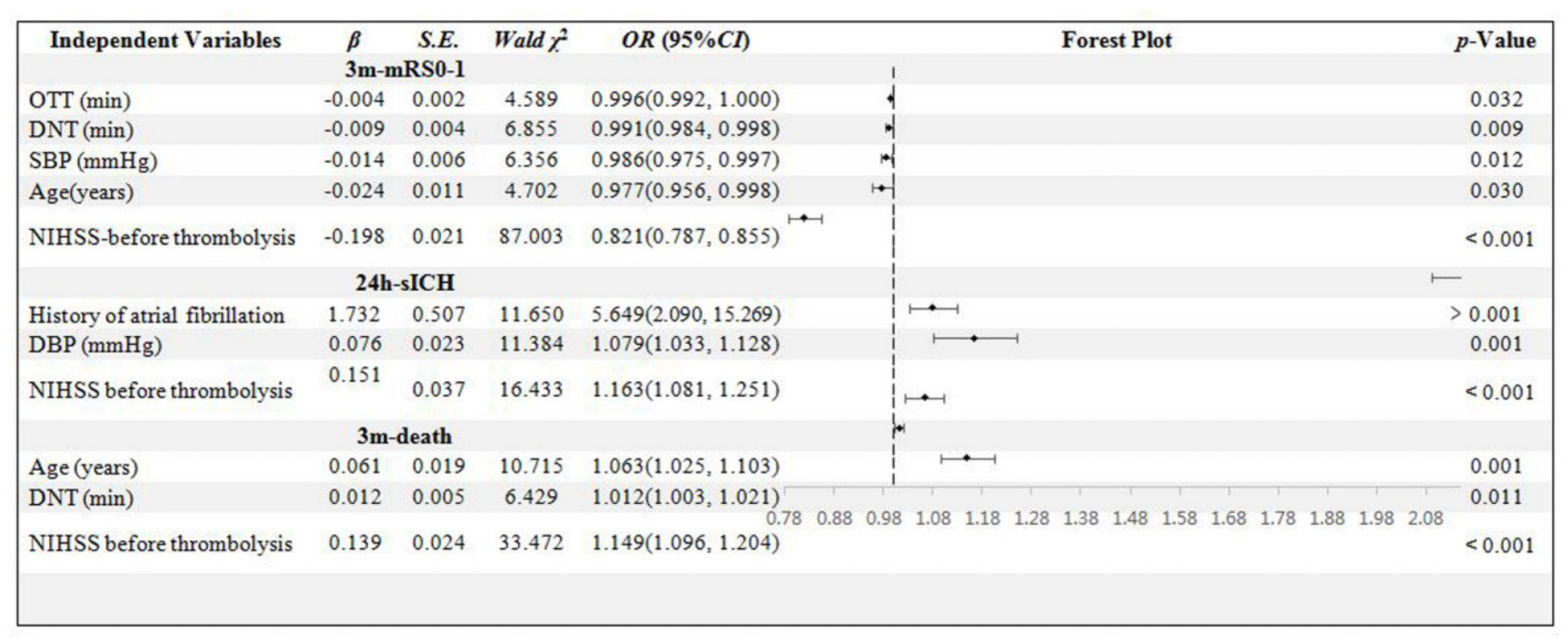
Binary logistic regression analysis in age < 80 years group. OTT, onset to thrombolysis time; DNT, door to needle time; SBP, systolic blood pressure; DBP, diastolic blood pressure; mRS, modified Rankin scale; sICH, symptomatic intracranial hemorrhage; NIHSS, National Institute of Health stroke scale.

In the age < 80 years sub-group, 486 patients' data were collected. The age, OTT, DNT, SBP, and NIHSS score were confirmed to be related to 3m-mRs0-1 independently (*p* = 0.030, 0.032, 0.009, 0.012, and < 0.001, respectively). The history of AF, DBP, and NIHSS scores were the independent influential factors to 24h-sICH (*p* = 0.001, 0.001, and < 0.001, respectively). Age, DNT, and NIHSS scores were significantly related to 3m-death (*p* = 0.001, 0.011, and < 0.001, respectively) (Figure 4).

## Discussion

The selection of r-tPA dosage of intravenous thrombolysis in AIS is still controversial. To resolve the dispute, many prospective studies (4–9, 13) preset drug dose coefficients, included patients according to the dose coefficient group, and compared the efficacy and safety of thrombolytic. But relative to the question of whether the dose of thrombolytic drugs should be standardized or individualized, maybe the real question the physicians are facing is: for individuals receiving thrombolytic therapy, should the given dose be the maximum safe dose that an individual can accept, or using dose titration to maximize the patients' benefits? Many physicians, when they treated AIS patients with thrombolytic therapy, still follow the “drug efficacy and safety” principle. Therefore, our study is retrospective and based on real clinical treatment activities. Physicians face many factors when deciding on a dose, based on things such as a patient's reaction or the cost of the treatment. Thus, the relationship between the dosage of r-tPA and the prognosis of patients in real AIS emergency aid is important.

In our study, we found that dose grouping was not an independently influential factor in the outcome of patients. The characteristics of the patients, especially the NIHSS score, admission glucose, and SBP still remained the most common independently influential factors to the prognosis, irrespective of the indicators, 3m-mRs0-1 or 24h-sICH and 3m-death. Other common influencing factors included the history of Af, DNT, OTT, diabetes history, and so on. The dose grouping had no significant effect on prognosis in our study.

Indeed, many trials have told us that the standard dose of thrombolytic drugs does not necessarily mean better efficacy, sometimes it does and sometimes it does not. However, in the same clinical trial, there are often significant differences in the comparison of efficacy and safety based on drug dose (9, 10, 14), and it is not consistent about the clinical efficacy and safety corresponding to the drug dose among trials. In our study, the dose coefficient had no independent and significant effect on outcomes, which required us to further refine the analysis. However, if we directly used a large set of data to analyze the correlation between drug dose coefficient and AIS outcome, we might lose the accurate description of the data. After all, no matter the guidance opinions or the clinical practice experience of physicians, the existence of such factors as old age (>80 years) or severe stroke itself would question whether thrombolysis should even be used (when DNT > 3 h), let alone the choice of drug dosage. This suggested that for AIS patients who are older than 80 years or younger, and whose NIHSS score is greater than or less than a certain critical value, we may need to consider differently about the dosage of r-tPA to reduce the risk of 24h-sICH or 3m-death.

The four subgroups in our study represented patients with four different characteristics. In NIHSS score ≥16 (severe patients) subgroup, MIOIF was DNT, which was correlated to 3m-mRS 0-1 and 3m-death. While in the subgroup of NIHSS score < 16, NIHSS was the MIOIF, which was correlated to 3m-mRS 0-1, 24h-sICH, and 3m-death. Multivariate logistic regression analyses showed that the drug dose coefficient was negatively correlated with 3m-death. Combined with the analysis of these two subgroups, we found that in AIS patients with a relatively mild neurological deficit, the higher dose coefficient would reduce 3-month mortality. While, when the nerve function was seriously damaged to a certain extent, the primary factor determining the outcome is DNT. In other words, we should give a standard dose of r-tPA to mild disability AIS patients. But in severe AIS patients, we need to minimize the DNT and give r-tPA to patients as soon as possible.

We used to consider that patients over 80 years of age might have a higher risk of bleeding after thrombolysis, and a lower dose might reduce such risk (1, 11). In the age ≥80 years subgroup, we were surprised to find that the main factor affecting the outcome of thrombolysis is not DNT or dosage, but diabetes history and NIHSS score on set. The results of this subgroup analysis seemed to highlight the risk of thrombolytic therapy for elderly AIS patients with diabetes, both with the severity at the stroke onset. However, such risk factors do not seem to be highlighted in thrombolytic guidelines (1, 11). This may be related to the relatively small sample size (51 cases) in our study. In future, based on the increasing number of patients, more analysis is be needed. In the subgroup of age < 80 years, the main factors influencing the outcome of thrombolytic therapy were DNT, age, NIHSS score, and also on.

### Mainstream factors

It should be noticed that the dose coefficient tended to affect the 3-month mortality, which was also negatively correlated to criticality.

Anyway, the limited sample size from the single-center database is the limitation of our study. Furthermore, there is a possibility of bias due to the different number of cases between groups when grouping. We are looking forward to enlarging the sample size to further clarify whether the dose coefficient is related to outcomes in r-tPA-treated AIS patients. The issue of r-tPA dosage and side effects will continue to be debated in future clinical work, especially in the use of special populations. Our study might provide a research direction for the dose selection of intravenous thrombolytic therapy for elderly and severe AIS patients.

## Conclusion

The non-standard dose group (0.6 mg/kg ≤ dose < 0.9 mg/kg) shows no difference in safety and effectiveness with the standard dose group (0.9 mg/kg) in our study, even in older (≥80 years) or sicker (NIHSS score ≥ 16) ones. While in patients with mild to moderate stroke, the dose reduction was significantly associated with 3-month death from our subgroup analysis. The standard dose should be considered first according to current evidence and AIS guidelines, but the non-standard dose (0.6 mg/kg ≤ dose < 0.9 mg/kg) might be an option in the actual diagnosis and treatment process considering the patient's clinical profile and financial condition.

## Data availability statement

The original contributions presented in the study are included in the article/supplementary material, further inquiries can be directed to the corresponding author.

## Ethics statement

The studies involving human participants were reviewed and approved by Ethics Committee of Shanghai Sixth People's Hospital. The patients/participants provided their written informed consent to participate in this study. Written informed consent was obtained from the individual(s) for the publication of any potentially identifiable images or data included in this article.

## Author contributions

JY and FW were involved in the design of the study and wrote the manuscript. JX and JD collected all the data. FZ checked the database. XZ and KL evaluated the results. RW was involved in the statistical analysis. FC and YZ evaluated the results and revised the manuscript. All authors have approved the final version of the manuscript.

## Funding

This research was supported by the Foundation of National Facility for Translational Medicine (Shanghai, TMSK-2020-108) and Science and Technology Commission of Shanghai Municipality (19411968500 and 19401972805).

## Conflict of interest

The authors declare that the research was conducted in the absence of any commercial or financial relationships that could be construed as a potential conflict of interest. The reviewer JS declared a shared affiliation with the authors to the handling editor at the time of review.

## Publisher's note

All claims expressed in this article are solely those of the authors and do not necessarily represent those of their affiliated organizations, or those of the publisher, the editors and the reviewers. Any product that may be evaluated in this article, or claim that may be made by its manufacturer, is not guaranteed or endorsed by the publisher.

## Abbreviations

r-tPA: Recombinant tissue plasminogen activator
3m-mRS0-1: 3 months mRS 0-1
24h-sICH: symptomatic intracranial hemorrhage within 24 h
NIHSS: National Institute of Health stroke scale
DNT: Door to needle time
MIOIF: the most important independent outcome-influential factor
AIS: acute ischemic stroke
sICH: symptomatic intracranial hemorrhage
mRS: modified Rankin scale scores
OTT: onset to thrombolysis time
SBP: systolic blood pressure
DBP: diastolic blood pressure
HTN: history of hypertension
DM: history of diabetes mellitus
CHD: history of coronary atherosclerotic heart disease
Af: history of atrial fibrillation
TIA: transient ischemic attack.
